# Tuning Secretomes for Regenerative Medicine

**DOI:** 10.3390/biology15120941

**Published:** 2026-06-16

**Authors:** Johanna Buschmann

**Affiliations:** Division of Plastic Surgery and Hand Surgery, University Hospital Zurich, Sternwartstrasse 14, 8091 Zurich, Switzerland; johanna.buschmann@usz.ch; Tel.: +41-44-255-98-95

**Keywords:** growth factor, cell-cell communication, wound healing, trophic factor, protein, extracellular vesicles

## Abstract

In this narrative review, the systematic tailoring of cell-derived secretomes for regenerative medicine is comprehensively discussed. The review begins by evaluating a diverse array of cell sources, including platelets, endothelial cells, osteoblasts, chondrocytes, tenocytes, and mesenchymal stem cells (MSCs). From this foundation, we critically analyze how modulating cell culture conditions can be used to directly influence the therapeutic and paracrine profiles of the resulting secretomes. Specifically, we address the impact of the following critical parameters on secretome fabrication: (i) Culture Medium Supplementation: The use of defined media, supplements, and chemical priming agents. (ii) Cell Cultivation Formats: A comparative analysis of 2D monolayer cultures versus 3D spheroids. (iii) Culture Duration and Fluidics: The effects of culture time alongside static versus dynamic culture systems. (iv) Microenvironmental Stress: The influence of varying oxygen levels (for example, hypoxia) and induced oxidative stress on secretory output.

## Simple Summary

The secretome of living cells consists of a unique array of factors and moieties released into their environment. These include bioactive proteins that drive cell–cell communication by eliciting specific biological responses. Such signaling pathways are vital for processes like wound healing and are governed by the secretome’s specific composition. This review highlights current methodologies for harvesting the secretome from different cell sources and from cells grown under different conditions, and discusses its applications in various areas of biomedical research, including regenerative medicine.

## 1. Introduction

During the last decades, the application of conditioned media (secretome) harvested from living cells and released into their supernatant culture medium has received increased attention [1,2,3,4,5]. Preclinical and clinical studies have demonstrated that the therapeutic benefits of cell transplantation are primarily driven by the paracrine effects of the secretome rather than by the cells themselves, or in the case of stem cells, by their differentiation potential. During cell transplantation, cell engraftment has been shown to be transient and low in many cases [6]. Consequently, cell-free secretome therapies have gained significant interest, as they allow for immediate clinical application without the logistical constraints of cell readiness in the desired amounts at a planned date [7].

Besides stem-cell-derived secretomes for regenerative medicine purposes, with their reported anti-inflammatory [8] and immunomodulating effects [9], other cell types or co-cultures have also been used to generate multiple kinds of cell-derived secretomes. Platelets [10], vascular endothelial cells [11] lymphatic endothelial cells [12], osteoblasts [13], chondrocytes [14], or adipocytes [15] have been reported to yield specific secretomes for regenerative medicine applications. Secretomes exhibit distinct compositions depending on the anatomical origin of the tissue from which the cells were harvested. Furthermore, their proteomic signatures are heavily influenced by the cultivation method [16] and the collection period, which is typically limited to 0.5 to 3 days. This short timeframe is essential, as serum-free culture is required to isolate a specific signature without triggering confounding survival or apoptotic signaling. Additionally, oxygenation conditions, such as hypoxia or oxidative stress, are critical parameters during production that can significantly alter the secretome’s composition [17,18].

Hence, this narrative review will cover the following seven aspects, all referring to the purpose of producing a distinct secretome with a specific profile that may be translated to regenerative medical applications and therapies:

1. Different cell types;
2. Co-cultures;
3. Culture medium;
4. Dynamics in secretome composition as a function of culture time;
5. Cell format; 2D versus 3D cultures, spheroids, organoids;
6. Hypoxia;
7. Oxidative stress.

## 2. Materials and Methods

The literature search to find publications for this narrative review covered key words of the six aspects mentioned at the end of the Introduction. By consulting PubMed, the Web of Knowledge/Web of Science or Google Scholar databases, roughly 97.5% (198 articles) of the papers in this review were found. The primary inclusion criterion required that papers directly addressed specific experimental parameters that influence secretome composition, for example, comparing co-culture-derived secretomes to their monoculture-derived counterparts, or comparing hypoxia cell culture-derived secretomes to secretomes collected under normoxia. The rest (5 articles) were found on other platforms, such as Embase, SCOPUS or SportDiscus. Papers ranging from the year 2000 up to the present were considered during the literature search.

## 3. Different Cell Types

While secretomes can be derived from any cultivable cell type by collecting the supernatant of the culture, we selected and focused on 10 specific cell types. Obviously, this selection is not exhaustive nor comprehensive, which is a limitation. However, the presented selection encompasses cells commonly investigated in regenerative medicine and related cell-free therapeutic approaches and research.

### 3.1. Platelets

Platelets (or thrombocytes) are small, disc-shaped cell fragments essential for blood clotting and tissue repair. They are produced in the bone marrow by megakaryocytes, which fragment to release thousands of platelets into the bloodstream. Activated platelets secrete adhesive proteins, matrix metalloproteinases (MMPs), Adenosine diphosphate (ADP), and Thromboxan A2 (TxA2) [19]. Alpha granules in platelets contain growth factors, such as vascular endothelial growth factor (VEGF), platelet-derived growth factor-BB (PDGF-BB) or transforming growth factor-β (TGF-β), necessary for proper wound healing, as well as clotting factor fibrinogen, which are released and are part of the secretome composition of platelets [20,21]. Furthermore, platelet delta granules contain ADP, Adenosine triphosphate (ATP), calcium and serotonin [22]. Platelet-rich plasma (PRP) is applied for numerous purposes within regenerative medicine [23,24,25,26,27,28,29]. Although varying production protocols make it difficult to compare results across studies, and there is an attempt to standardize [30], the efficacy of PRP relies largely on the release of the granules and their bioactive factors. This secretome-based stimulation and mechanism renders PRP an effective tool for regenerative therapies. Besides plasma, platelet-rich fibrin may also serve as a source of factors with paracrine action, supporting regeneration [31] (Table 1).

### 3.2. Endothelial Cells

Vascular endothelial cell (EC) culture to produce conditioned medium for regenerative purposes was described with a serum-free protocol and a stringent washing step to lower albumin levels before proteomic analysis by LC-MS/MS for rat ECs [62] and has been reported to improve endothelial barrier tightness when harvested from brain microvascular ECs [11]. The composition of progenitor endothelial cells has been summarized with microparticles and exosomes, the latter having distinct paracrine actions regarding survival, proliferation and tubulogenesis, as they increase VEGF levels as well as promote the expression of endothelial nitric oxide synthase (eNOS), which expresses nitric oxide (NO) [63] (Table 1). Furthermore, progenitor endothelial cells may also release mitochondria that, in turn, increase intracellular ATP levels after uptake by ECs and improve the tightness of their tight junctions [63]—ideal therapeutic effects after ischemic stroke.

Lymphatic endothelial cell (LEC) cultures have also been used to harvest and analyze their secretomes. In contrast to conditioned medium of vascular ECs, the LEC-derived secretome particularly impacts the recruitment of immune cells, tissue drainage and the microenvironment by paracrine action. Specifically, the LEC secretome includes CXCL12 and CCL21; VEGF-C and VEGF-D for lymph angiogenesis; PD-L1 (podoplanin); and extracellular vesicles (EVs) to be distributed to the whole lymphatic system and containing, for example, miRNA (miR-126) [64]. In regenerative medicine, the LEC-derived secretome has been utilized to study the behavior of osteoblasts because complex lymphatic anomalies (CLAs) are a set of rare diseases with unique osteopathic profiles. It was shown that the LEC-derived secretome inhibits osteoblast proliferation and differentiation [12], providing insights into mechanisms of CLAs. In another study, the LEC-derived secretome was considered to regenerate lymphatic vessels after damage or hypoplasia [65].

### 3.3. Osteoblasts

The osteoblast secretory function is important for the homeostasis of bones. Besides release of trace elements and Ca^2+^-deficient apatite, osteoblasts secrete collagens, proteoglycans, glycoproteins, cytokines, chemokines and growth factors, among them BMPs, M-CSF, RANKL, OPG, WNT5A, WNT16, GM-CSF, IGFs, IGFBPs, CXCL12, SCF, IL-7, and IL-6, as well as CCL5 [13] (Table 1). Hence, the secretome of osteoblasts includes a diverse set of factors whose composition changes drastically upon infection (osteomyelitis), as reported by Granata et al. [13]. The osteoblast-derived secretome regulates bone mineralization, remodeling and the coupling between bone formation and resorption by osteoclasts. Harvested from an osteoblast in vitro culture, the secretome can be used for bone tissue engineering, as an injectable therapeutic, or for the induction of osteogenesis in mesenchymal stem cells via BMPs, among others, where crosstalk between differentiation pathways is of central importance [66]. Moreover, the osteoblast-derived secretome has been tested for its suppressive function of tumor growth in breast cancer-associated bone metastasis [67].

### 3.4. Chondrocytes

The chondrocyte-derived secretome holds significant potential for enhancing hyaline cartilage regeneration due to its anti-inflammatory and anti-catabolic properties. Typical components of chondrocyte-derived secretomes are ECM-related structural entities such as collagens (type II), fibronectin, aggrecan and COMP (cartilage oligomeric protein) (Table 1); ana- and catabolic-related factors Chitinase-3-like protein 1 (CHI3L1/YKL-40), MMP-1, MMP-3 and TIMP-1; proteoglycans (biglycan, decorin, lumican or fibromodulin); proteoglycan link protein 1 (HPLN1), which is responsible for linking aggrecan to hyaluronic acid; and some IGFBPs [68]. While studies on the whole chondrocyte-derived secretome remain scarce, chondrocyte-derived extracellular vesicles (C-EVs) have been shown to effectively induce chondrogenic differentiation and enhance the proliferation of human umbilical cord-derived MSCs [69].

### 3.5. Adipocytes

Paracrine signaling of adipocytes has been investigated regarding the onset of diet-induced obesity [70] or obesity-induced chronic inflammation with an increased risk for cancer [71]. Under normal conditions, adipocytes secrete adiponectin, leptin, MMPs, protecting lipokines (like PAHSAs), resistin and ECM proteins such as collagen I; however, under obesity, the secretome turns pro-inflammatory with increased levels of TNF-α, IL-6, and IL-1β [72] (Table 1). Furthermore, the adipocyte-derived obesity secretome contains increased levels of free fatty acids and plasminogen activator inhibitor-1 (PAI-1). Besides these components, the main constituent of adipocyte-derived secretomes is EVs with multiple different cargo components, depending on the microenvironment and cellular crosstalk [73]. Typically, EVs from adipocytes of non-obesity fat tissue exhibit miRNAs that support insulin sensitivity, while EVs from adipocytes of obesity fat tissue contain miRNAs that induce inflammation in the liver and the muscles [74].

### 3.6. Tenocytes

The secreted factors of tendon cells may be useful as therapeutic support during tendon healing. However, tendon cells are characterized by a low metabolism, indicating a rather scarce secretome compared to professional secretory cells like MSCs. Nevertheless, a proteomic analysis has been reported by Marvin and co-workers, where they analyzed the “superhealer” Murphy Roths Large (MRL/MpJ) mice tendon-derived secretome and differentially compared its signature to “normal” standard Black-6 (B6) mice, both when collected under static and dynamic conditions. Tendons of MRL/MpJ mice are known to heal without scarring in a more regenerative way than normal tendons do [75]. Interestingly, the B6 secretome contained IL-3, in another notation multi-colony-stimulating factor (MCSF), while the superhealer did not [76]. While TNF-α was found only under dynamic culture conditions as a constituent of both B6- and superhealer-derived secretomes, TNF RII was only detected in the static secretomes—indicating an impact of further culture conditions on the secreted factors. Though not analyzing the secretome in detail, an experimental study using the secretome of tendon stem progenitor cells in a rat Achilles tendon partial defect model showed improved healing compared to the control [77]. Furthermore, Laurent et al. analyzed the whole fresh lysate of tendon progenitor cells (not the secretome) and analyzed their proteome, including MMP-2, sEGFR, TIMP-1 and MCSF as the most prominent constituents [78], which was considered to be an option for cytotherapy-inspired injectable preparations after functionalization with hydrogels [78] (Table 1).

### 3.7. Neural Cells

Neural cells release various neurotrophic factors, including brain-derived neurotrophic factor (BDNF), glial cell line-derived neurotrophic factor (GDNF), nerve growth factor (NGF), and neurotrophin-3 (NT-3) (Table 1). Research has demonstrated that the secretome of neural progenitors improves motor patterns in Parkinson’s disease rat models induced by 6-hydroxydopamine (6-OHDA), a neurotoxin that selectively destroys dopaminergic neurons [79]. Furthermore, the secretome derived from microglia exhibits neuroprotective effects against 6-OHDA-induced toxicity, a benefit largely attributed to the secretion of transforming growth factor-beta 2 (TGF-β2) [80]. Olfactory ensheathing cells (OECs) also secrete neurotrophic factors that support neuronal growth, as evidenced by in vitro studies using the dopamine-producing PC12 cell line under 6-OHDA stress [81]. Finally, glial cell line-derived secretomes containing GDNF have been shown to provide resistance against 6-OHDA-induced toxicity in neuronal cell lines under in vitro conditions [82]. Collectively, these examples illustrate that the neural cell-derived secretome possesses significant neuroprotective properties.

### 3.8. Immune Cells

The effects of immune cell-derived secretomes have been documented across various cell types. The immune cell-derived secretome has been analyzed for quiescent and primary Toll-like receptor 4-activated macrophages with a focus on differential proteomics. Activated macrophages secreted other factors, some of them with a 10,000-fold increase compared with quiescent macrophages [83]. In another study, macrophages released factors that played a substantial role in olanzapine-induced insulin resistance in adipocytes [84]. Furthermore, the secretome of alternatively activated macrophages (AAMs) has been shown to enhance tumor invasion, specifically by promoting the spread of high-grade serous ovarian cancer spheroids [85]. In addition, beneficial effects of lymphocyte-derived secretomes were reported, with evidence that the secretome suppressed the progression of osteosarcoma [86]. Also, the secretome of senescent monocytes has been shown to release secretory factors that act as biomarkers for the prediction of the clinical outcome in patients [87]. As for neutrophil-derived secretomes, particularly their EVs and neutrophil extracellular traps (NETs) [88] that enable adherence of platelets and other cells, this secretome has been shown to link inflammation and thrombosis [89]. Importantly, immune cells release interferons like interferon-γ (INF-γ) to act antivirally or antibacterially, or they may support cancer clearance during immune therapy [52].

### 3.9. Mesenchymal Stem Cells (MSCs)

Composed of a sophisticated milieu of soluble proteins, cytokines, growth factors, and EVs (Table 1), the MSC-derived secretome exerts multifaceted therapeutic effects. Rather than targeting a single isolated pathway, it acts through broad paracrine mechanisms to coordinate tissue repair and to support regenerative rather than fibrotic healing by promotion of wound closure and reduction of scar formation [90]. Key components such as VEGF, TGF-β, and various interleukins orchestrate simultaneous biological responses. They suppress chronic inflammation by modulating macrophage polarization, rescue damaged parenchymal cells from apoptosis, and stimulate robust angiogenesis to re-establish local blood supply. There is substantially more literature on secretomes harvested from undifferentiated MSCs as compared to the corresponding secretome from fully differentiated osteoblasts, chondrocytes or tenocytes, respectively. MSCs are typical secretory cells [2,5,9], with beneficial specific effects on allotransplantation [91,92], spinal cord injury healing [93], tendon regeneration [94,95], bone regeneration [96], lung repair [97], autoimmune diseases [8] and dermatitis [98,99]. Crucially, MSC-derived secretomes possess an inherent homeostatic versatility. When introduced into specific damaged microenvironments (whether in cutaneous wounds, ischemic cardiac tissue, or osteochondral defects), the secretome functions as a dynamic injury sensor. It delivers broadly acting trophic factors that mitigate acute tissue damage while establishing a receptive framework for endogenous stem cell recruitment and subsequent structural remodeling. Consequently, the MSC-derived secretome represents a scalable, readily standardized, and highly adaptable biotherapeutic toolkit capable of driving multi-lineage tissue regeneration.

Depending on the anatomical site from which the MSCs are harvested, the secretome composition varies [100]. Easily accessible fat tissue, particularly in plastic surgery after abdominal reduction plastics, is an MSC source that has been widely explored, and adipose-derived mesenchymal stem cells (ASCs) have been characterized and used for several preclinical and clinical modalities [101,102,103]. The bone marrow is another typical source for MSCs, called bone marrow-derived mesenchymal stem cells (BM-MSCs), for which many positive reports support their application in regenerative medicine [104,105,106], while others point to the often high levels of pro-inflammatory cytokines like IL-6 [107]. To treat Parkinson’s disease, the MSC-derived secretome has been reported to have positive effects, and amelioration strategies for secretome production have been reviewed [108]. Also, it has been shown that the secreted factors of adipose-derived stem cells exhibit synergistic features between secreted exosomes and secreted proteins in muscle regeneration [109]. Furthermore, dental MSC-derived secretomes, similarly to the bone marrow MSC-secretome, activate molecular and cellular mechanisms, which determine the effectiveness of cell-free therapy; a report by Bar et al. emphasized, for example, the positive effects in cartilage regeneration, potentially caused by IL-10 (Table 1), that reduced IL-1β, IL-6 and levels of TNF-α [3]. Then, the treatment of hair loss, or alopecia, was reviewed with respect to MSC-derived secretomes [29]. In this regard, particularly positive effects were attributed to MSC-derived EVs [29]. Noteworthy to mention, however, is the fact that MSC-derived secretomes, particularly exosomes, may lead to adverse impacts in cancer-related contexts. A study by Simão et al. showed that ASC-derived extracellular signaling factors promoted tumor plasticity and pro-tumoral behavior in ovarian cancer cells, and the bidirectional communication between ASCs and ovarian cancer cells was emphasized to alter the tumor microenvironment in favor of metastasis [110]. Crosstalk between MSCs and cancer cells has been reported to be controversial [111]. The main causes for this controversy lie in the type and the origin of the MSCs, cell culture conditions, exosome collections, stages and types of tumor in MSC-tumor crosstalk, composition of the tumor microenvironment, and the age of cell donors, among others [111]. Hence, depending on the context, secretomes may not only have positive regenerative effects but may also lead to a metastasis-prone environment.

### 3.10. Induced Pluripotent Stem Cells (iPSCs)

The secretome of induced pluripotent stem cells has been investigated in the context of radiation-induced damage to brain tissue. Distinct proangiogenic, pro-inflammatory and immunomodulatory factors have been identified, such as MCP-1, IL-6, IL-8, and ANG [112]. As a conclusive remark of their study, the authors judged iPSC-derived secretomes to be radioprotective; nevertheless, they mentioned that the potential to trigger quiescent cancer cells to proliferate is not to be neglected [112]. To examine whether iPSC-derived secretomes facilitate innate adaptive responses after lung tissue loss, adult dogs underwent surgical removal of one lung and were repeatedly administered iPSC-derived secretomes via inhalation. Dane and coworkers report that the inhalation of iPSC-derived secretomes enhanced a beneficial remodeling in the remaining lung, leading to improved lung function [113]. Other studies discuss the technical challenges of iPSC-derived secretomes, particularly during commitment towards distinct differentiated phenotypes, such as neurons, cardiomyocytes, fibroblasts or insulin-producing cells [114,115,116,117,118]. In sum, secretome components released by iPSCs exhibit a distinct regenerative potential.

### 3.11. Comparison of Secretomes Harvested from Different Cell Types

Although the inter-donor variability may be pronounced for any cell source with many subpopulations reported, for example, in adipose-derived stem cells [119], terminally differentiated cell types such as osteoblasts, chondrocytes, tenocytes or endothelial cells secrete, on average, fewer factors and in a lower amount than typical secretory cells like immune cells, thrombocytes or undifferentiated MSCs that maintain a vastly more robust and diverse secretome. Using a fully differentiated cell-derived secretome may have the advantage for the application of such a secretome during the regeneration of the organ from which it was originally harvested. The osteoblast-derived secretome, for example, specifically promotes bone regeneration, caused by factors like alkaline phosphatase, collagen I, osteocalcin, BMP-2, -4 and -7, osteopontin, periostin, VEGF, RANKL, or osteoprotegerin (OPG). While MSC-derived secretomes contain broadly acting trophic factors that support general bone regeneration, lineage-specific secretomes offer a highly specialized molecular profile that directly targets the precise signaling pathways of bone healing. For example, Kim et al. compared the secretome of undifferentiated human bone marrow-derived mesenchymal stem cells (hBMSCs) and differentiated osteoblasts by differential proteomics. They found that calcium homeostasis-related proteins were upregulated, whereas stem cell proliferation-related proteins were downregulated in osteoblasts during differentiation compared to undifferentiated hBMSCs [120]. Thus, a clear secretome shift occurred when the undifferentiated MSC-derived secretome profile was compared to the differentiated osteoblast-derived secretome in terms of protein composition. During the transition from multipotent stem cells to a specialized terminal lineage like osteoblasts, the cell’s energetic focus shifts from paracrine signaling to structural production (for example, collagen I for ECM regeneration). While the undifferentiated MSC-derived secretome focuses primarily on trophic, immunomodulatory, and pro-survival factors, where the MSC-derived secretome functions essentially as an injury sensor or paracrine factory pumping out massive amounts of cytokines (IL-6, IL-8), chemokines, and growth factors like VEGF, HGF, and TGF-β, the secretome of differentiated cells shifts toward specialized, dense ECM structural components (like Type I/II collagens, osteocalcin or aggrecan) on account of fewer signaling molecules. Also, the EVs change their cargo upon transition to a differentiated cell line. While the predominant protein cargo of EVs secreted from undifferentiated MSCs impacts biological functions like systemic cell recruitment, tissue homeostatic sensing, and localized immune suppression, the cargo content of differentiated cells covers the initiating localized hydroxyapatite crystal formation and calcium and phosphate ion concentrations (osteoblasts); the articular cartilage protection, metabolic cartilage homeostasis, and ECM synthesis (chondrocytes); or direct mechanical matrix remodeling, tendon fiber alignment, and localized fascial repair (tenocytes).

**Figure 1:**
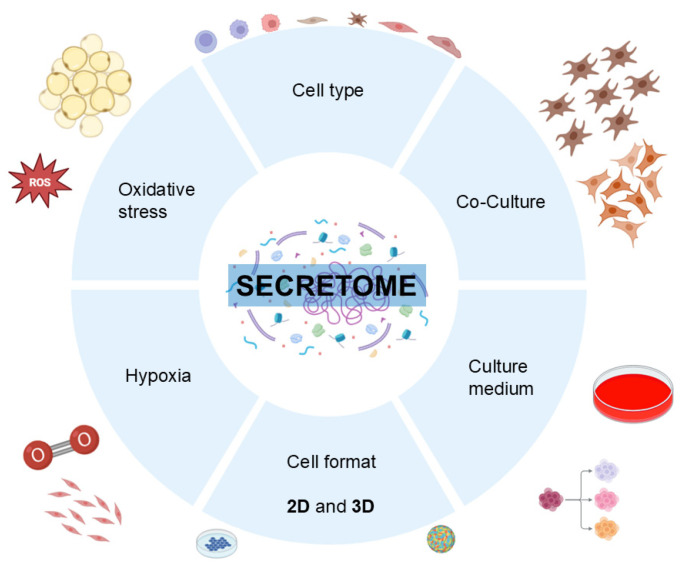
The schematic shows that different cell sources and phenotypes; a co-culture of different cell types; addition of supplements in the culture medium (for example, for stem cell differentiation); the cell format, such as 2D versus 3D spheroids; the oxygen tension; and the oxidative stress have an influence on the secretome that the cells release. The schematic was drawn with elements from BioRender^®^ (https://www.biorender.com/, accessed on 21 May 2026).

Consequently, the choice of cell type is a critical parameter when planning secretome production. However, additional factors, such as co-culture systems, medium supplementation, spheroid cultivation, oxygen levels, or the induction of oxidative stress, may further exert a pivotal influence on secretome composition (Figure 1).

## 4. Co-Culture-Derived Secretome

Although secretomes harvested from single cell types with approximately homogenous cell populations are attractive for regenerative medical processes, the secretome harvested from co-cultures may have advantages because cell-to-cell communication via paracrine factors represents one of the fundamental concepts in physiology and pathology [121,122,123,124]—and may offer compositions superior to those collected from mono cell culture. Hence, preclinical experiments using co-cultures are not only performed to study impacts of secreted factors from one cell type on the other cell type but may be harvested and applied for regenerative medicine purposes.

The platelet and MSC co-cultures’ secretome was reported to be acting beneficially in tendon-to-bone tissue engineering, where the combination of PRP and MSCs was also found to have positive effects [125]. Moreover, idiopathic pulmonary fibrosis (IPF) was studied by co-cultures of fibroblasts and immune cells of the adaptive and the innate immune systems, where secreted factors have been shown to influence and contribute to the fibrotic mechanisms of the IPF patients via cell–cell crosstalk [126]. Interestingly, the co-cultivation of rabbit ACL remnant cells with rabbit BMSCs resulted in the secretion of EVs that primarily increased the cell viability, proliferation, migration and gene expression of collagen synthesis- and TGF-β-, VEGF- and tenogenesis-related genes in both cell types, while EVs collected from BMSC culture alone did not exhibit such substantial effects [127]. The authors concluded that the coexistence of the two cell types provoked a distinct co-culture-derived secretome with beneficial effects for the maturation of the implanted graft in an ACL reconstruction model [127].

Another study investigated the crosstalk between macrophages (M0, M1, M2) and adipocytes to gain insight into the mechanisms underlying olanzapine-induced insulin resistance. Dipta et al. reported that the M2 macrophage-derived secretome triggered insulin resistance in adipocytes in an olanzapine-dependent manner. Notably, this induction of insulin resistance was even stronger than in cultures supplemented with pro-inflammatory cytokines (in the absence of M2 macrophages), indicating that other secretome components may act synergistically to promote insulin resistance [84]. Furthermore, the M2 macrophage-derived secretome has been shown to promote the disaggregation of ovarian cancer spheroids. Because of this complexity, it was suggested that targeting the JAK2/STAT3/MMP-9 pathway is more effective than inhibiting individual factors, as the collective components of the secretome drive metastasis [85].

In an in vitro study, a co-culture of human endothelial progenitor cells and MSCs showed that their secretome released paracrine factors that enhanced cell proliferation and angiogenesis, triggered through the PDGF and Notch signaling pathways. Such a secretome may be applied in wound healing, as it combines specific pro-angiogenic, immunomodulatory and mitogenic entities [128]. Moreover, a proteomic analysis where the secretome from a monoculture of rabbit ASCs was compared to a 3:1 co-culture of rabbit ASCs and rabbit Achilles tenocytes showed that, when applied to a tenocyte in vitro culture, both increased the *IL-6* gene expression, but the ASC-derived secretome did it ina more pronounced manner, which was judged as a disadvantage since it promotes inflammation. The co-culture-derived secretome was additionally convincing with its differentially upregulated proteins biglycan and tenascin-C, both tendon-related proteins, and was then rated superior for therapeutic application in tendon repair compared to the ASC-derived secretome [7].

Thus, the field of co-culture-derived secretomes is rapidly expanding. Though most published studies demonstrate that secretomes harvested from a cell-to-cell dialogue yield far superior therapeutic outcomes compared to monoculture secretomes, there are also exceptions. Kastner and colleagues report on a comparison of a hypoxic cardiomyocyte culture either co-cultivated with MSCs or treated with the secretome of an MSC monoculture [129]. They showed that cardiomyocytes treated with the MSC-derived secretome exhibited more pronounced changes in *HIF-1α* gene, *Rho A* gene and IL-18 protein expression than when directly co-cultured with MSCs, demonstrating first that the secretome changes upon co-culture and second that it was disadvantageous compared to an application of an MSC-monoculture-derived secretome. When applied as a regenerative therapy for myocardial ischemia, the choice of MSC-derived secretome was, for once, judged superior to that from a co-culture.

In sum, co-culture-derived secretomes represent a powerful, next-generation cell-free paradigm in regenerative medicine. By cultivating two or more distinct cell types together, researchers harness bidirectional paracrine signaling to generate a specialized, highly potent cocktail of biomolecules. This approach yields a significantly more therapeutic secretome, in most cases, than secretomes collected from single-cell monocultures.

## 5. Culture Medium

### 5.1. Modulation of Secretome by Culture Supplementation of Specific Factors

To treat Parkinson’s disease, cell-free approaches, including secretomes, are becoming increasingly attractive. Specifically, secretomes derived from embryonic stem cells, iPSCs, and MSCs have been shown to exert beneficial effects in this regard. Particularly interesting is the reported modulation of these secretomes by intracellular mechanisms or external cues, both triggered by cell culture medium supplementation [108]. For example, MSCs adapt to varying culture conditions; supplementing the culture medium drives distinct phenotypic commitments [130], which substantially alters the corresponding secretome. Furthermore, addition of cytokines like pro-inflammatory trophic factors modulates the MSC-derived secretome [131]. Finally, the application of two ASC-derived secretomes, where ASCs were cultivated either native (no supplements) or cytokine-supplemented in an osteoarthritis explant model, did not reveal differences—with both kinds of secretomes counteracting the upregulated MMP activity. However, the authors suggested optimizing the secretome composition by adding different cytokines or growth factors to the ASC culture medium in the outlook [132].

Additionally, cultivating MSCs in combination with different scaffold materials has been comprehensively reviewed and ultimately can modify their secretome [133]. A growing body of literature in biomaterials and tissue engineering confirms that scaffold physical properties, such as chemical composition, mechanical stiffness [134], and architectural dimensionality, directly determine the molecular profile of the cell secretome. When MSCs are cultured on varying scaffolds, their mechanoreceptors, such as integrins, translate physical cues into distinct biochemical outputs, profoundly shifting the concentrations of growth factors, cytokines, and EV cargos. Biochemical and biophysical characteristics of scaffolds for MSCs have been reviewed with respect to their influence on secretome composition [135].

### 5.2. Static Versus Dynamic Cultivation

**Figure 2:**
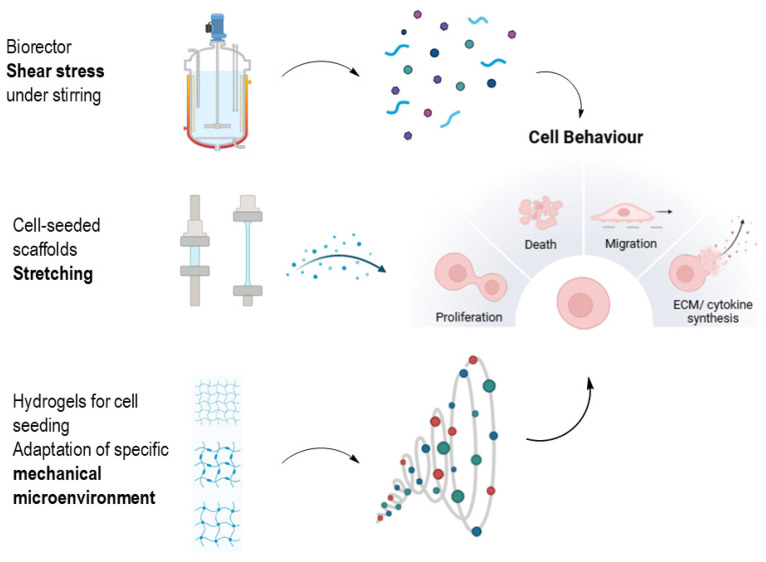
The cellular secretome varies upon mechanical stimulation. In bioreactors, shear stress regulates the composition of the secreted factors. Cell-seeded scaffolds may be stretched—another option to tune the secretome for regenerative purposes. Hydrogels with different stiffness may provoke different secretomes, which in turn influence cell behavior, such as proliferation, migration, ECM synthesis or cell death. The schematic was drawn with elements from BioRender^®^ (https://www.biorender.com/, accessed on 21 May 2026).

Whether the cell culture is performed under static or dynamic conditions (subjected to laminar or oscillatory fluid flow (shear stress) [136,137], compression [138], or stretching [139]), it will result in a completely different composition of secreted factors. This may offer a tailored production of a specific secretome envisioned for a therapeutic approach in regenerative medicine (Figure 2).

## 6. Dynamics in Secretome Composition as a Function of Culture Time

Another aspect of cell-derived secretome production is the dynamics of secretome composition depending on culture time. The composition of the secreted factors changes with culture time; in other words, the secretome is a function of how long cells are cultivated and whether they reach confluency. Indeed, the higher the confluency, the closer the cells are to each other, and whether it is a 2D monolayer or a 3D multilayer that is building up at later time points has a remarkable impact on the secretome that the cells release into their environment. In the first 3–5 days, proliferation, cell migration, and protein synthesis enhancing factors are normally released [7], and the secretome shifts towards higher concentrations of angiogenic factors, cytokines, and extracellular matrix (ECM)-remodeling proteins, with growth factor release peak at 7–10 days. After confluency, and particularly after repeated passaging, cells may enter a senescent phase, where they get into the senescence-associated secretory phenotype [140,141], and their secretome is characterized by increased levels of pro-inflammatory cytokines, MMPs, LOX, reactive oxygen species (ROS) [142] and β-galactosidase [46]—representing a completely different image than at early passages. In a study by Kastner et al., a timely dynamic change of ischemia and regeneration-related genes expressed by cardiomyocytes was reported, where the time course for specific marker genes was different between a hypoxic cardiomyocyte culture treated with an MSC-derived secretome compared with a co-culture of cardiomyocytes and MSCs [129]. Secreted pro-inflammatory IL-18 protein expression varied substantially at 4, 8, 24, 48 and 72 h for all different conditions examined, i.e., normoxic cardiomyocytes, normoxic MSCs, hypoxic cardiomyocytes, hypoxic cardiomyocytes treated with an MSC-derived secretome and the hypoxic co-culture of MSCs and cardiomyocytes, respectively [129]. In another report, a time-resolved proteomic atlas of hepatocyte, myocyte, pericyte and myeloid cell-derived secretomes was generated by direct purification of biotinylated secreted proteins from blood plasma in mice. In addition, the authors uncovered a dynamic and new nutrient-dependent reprogramming of the hepatocyte secretome [143]. Furthermore, to investigate the temporal dynamics of paracrine factors, the MSC-derived secretome was collected at defined intervals (1, 2, 3, or 4 days of cultivation) and subsequently applied to in vitro cultures of neurons and glial cells. This revealed that the biological effects of the secretome were time-dependent. Both neuronal and glial viability were differentially modulated, showing a direct correlation with the specific harvesting time point, although an in-depth analysis of secretome composition was not provided [144]. Therefore, the culture time for secretome production belongs to one of the fundamental conditions to be considered in the design parameters.

## 7. Cell Format

A further important condition is the cell format. Depending on whether cells are cultivated in a 2D monolayer, in a 3D spheroid format, as an organoid or seeded on a 3D scaffold material [145], the secretome will vary with respect to composition [146,147]; this offers another parameter that can be varied during secretome production. Specifically, corneal wound healing was investigated under either secretome harvested from a 2D BMSC culture or a corresponding 3D culture within an electrospun PCL scaffold [146]. The authors reported a differential secretome composition as assessed by multiplex assay and found that the secretome released from the 3D format led to lower α-SMA expression, a typical indicator for scar formation during wound healing [146]. Another study reports on the release of angiogenic and wound healing-related factors from spheroids of human ASCs entrapped in a 3D-printed/electrospun alginate scaffold [148]. The regenerative potential of lung spheroid cell secretome has furthermore been shown by an experiment of inhalation to treat different models of lung injury and fibrosis, resulting in the reestablishment of the normal alveolar structure and a decrease of both collagen accumulation and myofibroblast proliferation, the latter representing two hallmarks of regenerative healing [149]. Evidence of the compositional divergence between 2D and 3D MSC-derived secretomes is further demonstrated by their differential immunomodulatory activity against in vitro macrophage cell culture. Specifically, secretomes derived from a 3D spinner flask culture outperformed those harvested from 2D monolayers by delivering a substantially enhanced and more potent cocktail of anti-inflammatory trophic factors [150]. For the treatment of atopic dermatitis, secretomes harvested from 3D cultures significantly outperformed conventional 2D monolayer configurations [151]. This approach yielded markedly enhanced keratinocyte sheet formation and superior wound healing outcomes [152,153,154].

## 8. Hypoxia

Normal air has an oxygen tension of approximately 21%. However, in the human body and depending on which organ is under view, oxygen tension is substantially lower, such as in arterial blood (13.2%), kidney (9.5%), lung (5.6%), liver (5.4%), brain (4.4%) [155] or tumors (2%) [156]. Hence, in vitro cell cultures under 21% do not actually reflect in vivo conditions, which led researchers to design experiments with hypoxia-induced cell-derived secretomes under different oxygen tensions, resulting in secretomes of different composition [18,157,158,159].

For example, Ding and co-workers compared the effects of apoptotic EVs harvested from apoptotic stem cells previously grown under hypoxia and regular apoptotic EVs on cartilage repair in a rat osteochondral model. Hypoxia apoptotic EVs significantly boosted chondrocyte proliferation and migration and promoted M2 polarization in macrophages more effectively than standard apoptotic EVs, indicating differential composition of these EVs because of hypoxia [160]. A further study delineates the more prominent pro-angiogenic effects of secretomes produced under hypoxia in an iPSC culture compared to normoxia [161]. Also, cardiomyocyte proliferation was more pronounced under supplementation of a hypoxia-induced amniotic stem cell secretome compared to a secretome harvested under normoxia [162]. In another cardiomyocyte study, hypoxic culture conditions led to a doubled IL-18 protein expression after 24 h compared to the cardiomyocyte normoxic culture [129]. Furthermore, B-cell maturation antigen was reduced in melanoma cultures under hypoxia, along with less protein secretion compared with normoxia. Specifically, the hypoxic melanoma secretome significantly impaired CAR T-cell killing, which was caused by the secretion of EVs with a hypoxia-induced RNA signature involved in immunomodulation [163]. It has additionally been reported that hypoxia-cultivated MSCs and alkaline water mediate oxidative stress and inflammation in diabetic rats [164]. Stella et al. discussed new therapeutic strategies based on the genetic profile of mesothelial pleural mesothelioma and its interaction with the surrounding hypoxic microenvironment. They highlighted transcripts and microvesicles, which offer insights into the disease’s development and provide promising, actionable targets [165]. These examples underline a clear pro-angiogenic and anti-inflammatory signature of secretomes harvested under hypoxic cell culture conditions and show that secretomes released under hypoxia may help to elucidate mechanistic pathways.

## 9. Oxidative Stress

Oxidative stress may lead to epigenetic alterations in stem cells, resulting in differentially composed secretomes [166]. For example, cultivation of MSCs under oxidative stress has been reported to induce an antioxidant secretome; the oxidative stress primes the cells to secrete a rich array of proteins, cytokines, growth factors and exosomes, enhancing the antioxidant capacity of the MSCs to react against the oxidative stress and to mitigate it [167]. Additionally, cells grown under oxidative stress undergo a pro-inflammatory switch that results in a secretome with pronounced pro-inflammatory cytokine content. Then, the released vesicles and exosomes change their cargos, particularly the miRNAs. For example, Alibrandi et al. report that the miRNAs released to the surroundings are substantially altered under oxidative stress—and that the blood-derived secretome has the ability to alter the miRNA release from pro-inflammatory towards a more regenerative type [168]. It has to be emphasized, however, that low-level oxidative stress leads to different MSC-derived secretome alterations than high-level oxidative stress does. While low-level oxidative stress is reported to enhance cytoprotective factors, antioxidant proteins and pro-survival factors, high-level oxidative stress causes a harmful pro-apoptotic shift in the secretome composition, with inflammatory cytokines leading to cytotoxic effects and limited regeneration [167]. ROS preconditioning has therefore been reported for MSCs with dose–response curves, where supplementation of the culture medium with 25 μM hydrogen peroxide was judged as a favorite because it induced better secretome than the other tested concentrations. Such hydrogen peroxide preconditioned MSC-derived secretome contained more SOD, CAT and GSH-Px, induced by increased gene expressions of *Nrf2*, *HO-1* and *NQO-1* [169].

## 10. Characterization of Secretomes

To analyze and characterize a cell-derived secretome, a combination of proteomic profiling [170], quantification, and vesicle characterization techniques is used. Because the secretome consists of soluble proteins, nucleic acids, lipids and EVs, we address three main categories here.

### 10.1. Analysis of All Secretome Proteins: Proteomics

The gold standard for an unbiased, large-scale identification and quantification of all proteins in a secretome is mass spectrometry (MS) [171]. Liquid chromatography (LC) can support an in-depth MS analysis. The LC-MS/MS couples LC with tandem mass spectrometry in order to sequence and identify complex protein mixtures. Either label-free or chemical labeling can be used. While the label-free approach compares protein spectral intensities across different samples without chemical labels, the chemical labeling one uses isobaric tags to multiplex up to 16 samples in a single MS run for precise relative quantification. Also, affinity proteomics are frequently used [170]. Furthermore, metabolic labeling incorporates heavy amino acids into live cells to distinguish their newly secreted proteins from background serum proteins [172].

### 10.2. Analysis of Specific Proteins in the Secretome

Besides MS, targeted protein analysis can be applied, such as ELISA to quantify a single specific protein of interest in the secretome, or Multiplex Bead Arrays (Luminex) that uses color-coded beads to measure dozens of different cytokines simultaneously from a tiny secretome volume [173]. Also, the Western blotting technique is used in order to confirm the presence and molecular weight of a specific target protein, utilizing gel electrophoresis.

### 10.3. Analysis of Extracellular Vesicles

The analysis of EVs requires other analytical techniques. Since a major component of the secretome is housed within EVs, specialized tools are required to analyze them. Here, nanoparticle tracking uses light scattering and Brownian motion to determine EV size distribution and concentration [174]. To provide high-resolution visual imaging and to confirm EV morphology and structure, transmission electron microscopy is used. In addition, surface markers of EVs can be detected with nano flow cytometry, where profiles of CD63, CD81 or CD9 on individual extracellular vesicles can be quantified [175]. In accordance with the MISEV2023 guidelines [176], recognized separation techniques also encompass differential ultracentrifugation, density gradients or cushions, size-exclusion chromatography [177], fluid flow-based separation, and charge- or molecular recognition-based approaches. Nevertheless, every method presents inherent limitations, making a universally optimal choice difficult.

### 10.4. EV Delivery Approaches and Clinical Applications

Delivery approaches for EVs cover both direct systemic administration, where the resulting biodistribution has to be evaluated, as well as localized injections or scaffold-based sustained release options. All methods aim to protect the vesicles from rapid degradation, maximize their tissue-specific targeting and effects, and ensure a controlled release of bioactive molecules directly to the site of injury or lesion [178]. The resulting biodistribution represents a function of the administration mode, where intravenous injection, peritoneal injection, oral administration, intranasal administration, subcutaneous injection, inhalation, intra-tumor injection, intramuscular injection and other in situ applications were carefully reviewed by Su et al. [179]. Biomaterial-based sustained release systems were explored for ASC-derived EVs in combination with hydrogels. Such developments enabled better retention of the EV cargo in inflamed areas [180]. Other systems are based on 3D printing, where EVs were integrated directly into solid porous scaffolds to guide structural tissue remodeling [181].

Clinical applications of EVs have been reported for the following indications: prostate cancer [182,183], osteoarthritis [184,185], regulation of the innate immune system [186], periodontal regeneration [187], vascular diseases [188], breast cancer [189], ovarian cancer [190] and urological cancer [191]. The cell sources for harvesting EVs vary across these referenced studies, and so does their composition. The clinical application of EV-based therapeutics still has challenges, such as questions on how to choose the favorite cell source, harvesting techniques, accurate characterization or how to avoid undesirable off-target effects [192,193]. Nevertheless, EVs are among the most promising components of cell-derived secretomes for clinical translation.

## 11. Major Preclinical and Clinical Applications of Secretomes

The major clinical trials and preclinical studies are summarized in Table 2 and references therein.

## 12. Challenges and Future Directions

Although many beneficial effects have been attributed to cell-derived secretomes in regenerative medicine applications, there are several challenges to be considered. One of them is the reproducibility of secretome production, with batch-to-batch variability. Because cell sources exhibit a natural heterogeneity, which is not only pronounced for similar tissues collected at different sites (such as fat tissue for the collection of ASCs to produce ASC-derived secretome, where abdominal fat, fat from the back, or eyelid fat may vary with respect to their stem cell subpopulations) but is also manifest when different donors are compared. The production of a specific secretome utilizing dedicated purification and concentration protocols may lead to (slightly) different secretome composition. Given these facts, up-scaling secretome production may become difficult. During production, the percentage of serum proteins added to the culture medium may vary; also, cell expansion, cell passage number and confluency, the conditioning period, the cell culture medium and some microenvironmental cues may influence the secretome composition. There is still a big lack of standardization, although attempts in this direction have been made in defining Good Manufacturing Practice (GMP) baselines. For example, Chouaib and co-workers have established a GMP-compliant protocol for the production of MSC-derived secretome [200]. Consequently, regulatory demands and classifications have to be considered during secretome production, characterization, preclinical proof of efficacy and stability tests [201].

Another challenge is the choice of the delivery system, as different chemical compositions of vehicles may retain the various components of the secretome differently. Given the diverse intermolecular interactions between the secretome components and the delivery device, with secretome components exhibiting hydrophobic or hydrophilic moieties or hydrogen bonding, a controlled release for the whole secretome seems to be complex. Also, there are many secretome components in very low concentrations, rendering proper chemical analysis and quantification difficult. Furthermore, proper storage conditions (temperature and period) have to be evaluated and compared in an attempt not to alter or completely lose the bioactivity of the secretome [202,203]. Moreover, with respect to secretome application in the (pre-)clinical setting, dosage standardization is a challenge because in different animal models, different amounts of secretomes are applied—making comparison between different secretome studies or trials difficult.

Hence, analytical methods should be optimized to accurately characterize the secretome in the future. Moreover, pooled donor approaches should be established, and standardized purification protocols should be established. Dosages and administration modalities should be standardized, and regulatory demands and classifications should be defined and considered.

## 13. Conclusions

This narrative review highlights the key parameters influencing the composition of cell-derived secretomes, as illustrated by recent studies. A fundamental aspect is the specific cell source. While MSCs and platelets are highly secretory and heavily featured in the literature, osteoblasts and chondrocytes yield lower secretome quantities and are consequently less studied. Beyond these common sources, we review secretome profiles from blood and lymphatic endothelial cells, adipocytes, tenocytes, neural cells, and immune cells. Additionally, culture conditions represent a pivotal domain for modulating secretome composition. Critical parameters, including culture media and supplements, static versus dynamic cultivation, 2D versus 3D systems, cultivation time and its compositional dynamics, oxygen tension, and oxidative stress serve as powerful tools to tailor specific secretomes for applications in regenerative medicine. For translation into daily clinical practice, however, major hurdles are the reproducibility of secretome production, GMP-compliant manufacturing, proper storage conditions, analytical detection limits, standardized dosages and administration modes—all of which have to meet regulatory demands that have to be defined.

## Figures and Tables

**Table 1:** Different cell sources and their major secretome components; possible regenerative medical applications, with exemplary pathologies.

*Cell Source*	*Major Secretome Components*	*Biological Function*	*Application in Regenerative Medicine*
Thrombocytes	TGF-β	ECM regulation; collagen synthesis	Tendon repair [23,24]
	VEGF	Promotes angiogenesis	Bone repair [32]
	Fibrinogen	Converted to fibrin: clotting	As antimicrobials [33]
Endothelial cells	Nitric oxide (NO)	Vasodilation prevents clotting	Ischemic stroke [34]
	vWF	Platelet adhesion, blood clotting	Bleeding disorder [35]
Osteoblasts	Collagen I	ECM synthesis	Bone repair [36]
	Osteocalcin	Binding Ca and hydroxyapatite	Cranial bone repair [37]
	BMP-2	Osteogenesis and angiogenesis	Bone repair [38] Cartilage repair [39]
Chondrocytes	Collagen II	ECM synthesis	Cartilage regeneration [40]
	Aggrecan	Water uptake	Central nervous system [41]
	COMP	Stabilizing collagen network of ECM	Chondrogenesis [42]
Adipocytes	Adiponectin	Anti-inflammatory and tissue repairing	Enhancing MSC effects [43]
	Leptin	Regulation of energy	Nerve regeneration [44]
	IL-6	Dual: Pro-inflammatory and regenerative	Muscle regeneration [45]
Tenocytes	Collagen I	ECM synthesis	Tendon regeneration [46]
	Tenascin C	Provisional matrix wound healing	Wound healing [47]
	MMPs	Tissue remodeling	Muscle fibrosis [48]
Neural cells	BDNF	Neuroprotection	Neurological diseases [49]
	NGF	Axonogenesis	Eye diseases [50]
	IGF-1	Peripheral metabolism	Multiple sclerosis [51]
Immune cells	INFs	Immune cell activation; antiviral and/or antibacterial	Immunotherapy, cancer treatment [52]
	Perforin	Pore-forming; cytotoxic	Immunotherapy [53]
	TNF-α	Immune activation and inflammation	Pretreatment of hASCs for liver injury [54]
Mesenchymal stem cells (MSCs)	VEGF	Pro-angiogenic	Burn wound healing [55]
	HGF	Mitogen; cell survival	Lung and liver fibrosis [56]
	PGE2	Anti-inflammatory; M1-M2 shift; activation of stem cells	Tissue regeneration [57]
	IL-10	Anti-inflammatory; M1-M2 shift	Muscle regeneration [58]
	Galectins	Control autoimmune reactions; suppress T and NK cell proliferation	Cardiomyopathy [59]
	EVs or exosomes	Cargo may vary; protective miRNAs	Support angiogenesis in wound healing [60] Neurocognitive disorders [61]

**Table 2:** Different cell sources used for different medical indications, their study type and key outcomes.

*Medical Field/Indication*	*Study Type*	*Cell Source for Secretome*	*Key Therapeutic Outcomes and Findings*	*Reference*
Pulmonary Medicine	**Clinical Trial** (Phase II, RCT, 102 patients)	Bone marrow MSCs	Intravenous infusion showed improved survival rates and oxygenation; safely reduced severe respiratory inflammation	[194]
Cardiovascular Medicine	**Clinical Trial** (Phase I, Dose-escalation)	Umbilical cord matrix MSCs	Evaluated safety of targeted intra-articular injections using small extracellular vesicles manufactured under strict GMP constraints	[195]
Dermatology and Aesthetics (Skin Rejuvenation, Scarring, Hair Loss)	**Clinical Systematic Review** (17 clinical studies up to 2024)	ASCs and BMSCs	Confirmed favorable visual improvements in tissue remodeling, scar reduction, and hair follicle activation through topical/subcutaneous delivery	[196]
Neurology (Alzheimer’s Disease)	**Preclinical Systematic Review** (21 in vivo rodent studies)	Neural cells and MSCs	Significantly reduced amyloid plaque accumulation, suppressed reactive gliosis, and enhanced hippocampal neuronal density	[197]
Infectious Disease (Bacterial Infections & Sepsis)	**Preclinical Systematic Review** (37 in vivo rodent models)	BMSCs	Boosted host immune responses, decreased bacterial load in tissue, and improved overall survival rates in acute systemic infection	[198]
Oncology (Anticancer Therapeutics)	**Preclinical In Vivo Models** (Rodent tumor models)	Wharton’s jelly MSCs	Inflammatory-primed or engineered EV platforms resulted in 55–85% inhibition of tumor growth and cell migration across breast/lung model	[199]

## Data Availability

This review does not include data.

## Abbreviations

The following abbreviations are used in this manuscript:

2D	Two-dimensional
3D	Three-dimensional
AAM	Alternatively activated macrophages
ADP	Adenosine diphosphate
ATP	Adenosine triphosphate
ASCs	Adipose-derived stem cells
ANG	Angiopoietin
α-SMA	Alpha smooth muscle actin
B6	Black 6 mice (C57BL/6)
BMPs	Bone morphogenetic proteins
BDNF	Brain-derived neurotrophic factor
BM-MSCs	Bone marrow-derived mesenchymal stem cells
CXCL12	CXC motif chemokine 12 or stromal cell-derived factor 1
CCL21	CC motif chemokine ligand 21
CLAs	Complex lymphatic anomalies
CSF	Colony stimulating factor
CCL-5	CC motif chemokine ligand 5
COMP	Cartilage oligomeric protein
C-EVs	Cartilage extracellular vesicles
CDNF	Cerebral dopamine neurotrophic factor
ECs	Endothelial cells
ECM	Extracellular matrix
eNOS	Endothelial nitric oxide synthase
EVs	Extracellular vesicles
HPLN	Proteoglycan link protein 1
IGFs	Insulin like growth factors
IGFBPs	Insulin like growth factor binding proteins
IL-3; IL-6; IL-7; IL-8	Interleukin-3; -6; -7; -8;
iPSCs	Induced pluripotent stem cells
IPF	Idiopathic pulmonary fibrosis
JAK/Stat3/MMP-9	Januskinase/Signal Transducer and Activator of Transcription 3/Matrix-Metalloproteinase 9 pathway
LC-MS/MS	Liquid chromatography mass spectrometry/mass spectrometry
LECs	Lymphatic endothelial cells
LOX	Lysyl oxidase
MSCs	Mesenchymal stem cells
MMPs	Matrix metalloproteinases
M-CSF	Macrophage colony-stimulating factor
miRNA	Micro RNAs
MRL/Mpj	Murphy Roths Large mice
MCP-1	Monocyte Chemoattractant Protein-1
M2	Macrophages polarized M2 (alternatively activated macrophages)
NGF	Nerve growth factor
NT-3	Neurotrophin-3
NETs	Neutrophil extracellular traps
NO	Nitric oxide
OPG	Osteoprotegerin
OHDA	Hydroxydopamine
OECs	Olfactory ensheathing cells
PRP	Platelet-rich plasma
PD-L1	Programmed Cell Death 1 Ligand 1
PAHSAs	Protecting lipokines
PAI-1	Plasminogen activator inhibitor-1
PC12	Dopamine-producing cell line
RANKL	RANK ligand
ROS	Reactive oxygen species
SCF	Stem cell factor
sEGFR	Soluble epidermal growth factor receptor
TxA2	Thromboxan A2
TIMP-1	Tissue inhibitor of matrix metalloproteinase-1
TNF-α	Tumor necrosis factor alpha
TGF-β1; TGF-β2	Transforming growth factor beta 1 and 2
VEGF	Vascular endothelial growth factor
VEGF-C	Vascular endothelial growth factor-C
VEGF-D	Vascular endothelial growth factor-D
WNT5A	Wingless-related integration site protein 5A
WNT16	Wingless-related integration site protein 16
